# Therapeutical and Pharmaceutical Notes

**Published:** 1900-05

**Authors:** W. J. Martin

**Affiliations:** Kankakee, Ill.


### THERAPEUTICAL AND PHARMACEUTICAL NOTES.

UNDER THE DIRECTION OF W. J. MARTIN, M.D.C.,
KANKAKEE, ILL.

AGE OF TINCTURE OF IODINE. The time-honored injunction, to see that this tincture has been freshly prepared, seems to be in danger of joining the group of aphorisms so common in medicine and pharmacy, which is fast passing into a state of "innocuous desuetude." Recent experiments with this tincture have demonstrated that a tincture of long standing produced a deeper and more permanent stain on the skin than one of a recent make. Hence, the older the preparation the deeper and more lasting were the therapeutic effects. It was also demonstrated that instead of decreasing with age the tendency was in a directly opposite direction. This is accounted for, according to Merck's *Report*, "Because while iodine is quite volatile it does not vaporize with the rapidity of the alcohol in which it is dissolved to make the tincture."

ADULTERATION OF COCA LEAVES. Owing to the extreme scarcity and high price of Bolivian coca leaves, samples of this drug have been found on the market in a highly adulterated condition. Small jaborandi leaves is the adulterant mostly used for this purpose. Some of the samples of coca leaves examined were found to contain as high as 40 or 50 per cent. of jaborandi leaves.

USE AND ABUSE OF POULTICES. The injudicious use of the poultice has brought it into such general disrepute that its benefits are now oftentimes lost sight of and its use discarded. Dr. S. E. Sharp (*New York Medical Journal*) points out that the active principles of the poultice are moisture and heat, and hence it matters little what they consist of except so far as their capability of retaining heat is concerned. They must be generous in size, applied hot, and frequently changed. In general, it is better to use antiseptic fomentations upon open wounds, but the charcoal poultice is of considerable value as a deodorant in some cases of foul ulcer. The virtues of a poultice are seen in its power (1) to relieve congestion; (2) to promote absorption, favor resolution, and hasten suppuration; (3) to diminish tension; (4) to soften incrustations; (5) to stimulate healthy granulation, and (6) to perform the office of a deodorant.

Danger in Using Spider Webs as a Hæmostatic. On August 22, 1899, I was consulted by one of my neighbors with regard to a horse of his which had wounded itself with brambles surrounding a field it was in. I advised him to apply bandages soaked in creosolated water in order to stop the hemorrhage, which he said was violent. Three hours later I examined the wounds, which were in the forearm and knee. They were not bleeding, were not deep, and not serious. The bleeding had been stopped by applying spider webs, which, proving insufficient, had been supplemented by bands of cotton. I remarked that cobwebs were not advisable, and were even dangerous. I do not know whether my neighbor was convinced or not, for this old practice is very difficult to uproot. At any rate, I disinfected the wounds and sewed the edges together, promising a rapid cure.

Four days later the proprietor of the animal arrived in a state of consternation. He informed me that the beast was unable to walk, its limbs were swollen, and that it would not eat. I believed he was exaggerating, but I had to change my opinion when I saw the horse. Its attitude was one of great dulness. Its forelimbs were swollen from the elbow to the coronet, and moved in a "scythe-like" fashion. The skin, which was hot and painful to the touch, was slightly œdematous. In certain places little papules exuded viscous liquid without any smell. Not knowing what to attribute this complication to, I removed the pins, fearing infection in the wounds. I was reassured by their color and the nature of the pus which came from them.

Two days later the hind legs were all out in an eruption; also some spots of horsepox had appeared on the lips and on the nostrils, leaving no doubt as to their exact nature. Where did this horsepox come from? There were cows in the place. I questioned very sternly the persons who had had charge of the horse, but could get nothing out of them to satisfy myself. All that I could learn was that similar pustules always appeared on the udders of newly arrived cows, but that all these latter were healthy at that time.

I then asked where the spider webs had come from. They came from the byre, whose ceiling, by the way, is still thickly coated with them. That reply at once showed me the probable origin of this inoculation. The vaccine dust retained by the webs had been transmitted to the wounds, which had proved to be doors of admission for it.

The long period of uselessness of the horse which resulted from the inoculation was sufficient to convince the owner of the danger run in using spider webs as a hæmostatic, and this case was useful as an experiment, as it had more effect on him than any number of arguments would have had.—(M. Pecus, in the *Recuiel de Med. Vet.*)—*Veterinary Journal.*

ANTITETANIC SERUM AS A PROPHYLACTIC. It has been shown that guinea-pigs and other animals inoculated with the poison of tetanus survive when treated at once with antitetanic serum. In France, Nocard observed 375 animals of various kinds, all of which had been wounded accidentally or surgically, and subjected to tetanic infection. These animals were given antitetanic serum at once, before the disease had time to develop. As a result, not a single case of tetanus occurred among them. On the other hand, he noticed fifty-five traumatized animals that had been exposed to tetanic infection, every one of which developed the disease.

In the August number of *Medicine* Professor George F. Butler, M.D., says: "Dr. Joseph Hughes, one of the most eminent and conservative veterinary surgeons in Chicago, has used the serum as a prophylactic in over 500 cases following wounds, both surgical and accidental." Not a single case of tetanus has developed, though Dr. Hughes has used the serum where, by former experiences, he was justified in expecting the disease to manifest itself.—*Bull. Pharmacy.*

ANTISTREPTOCOCCIC SERUM. Denys (*Centralblatt für Chirurgie*, 1900, No. 3), after an experimental research on animals, conducted with streptococci of apparently one species, which were derived from a glandular abscess, employed his antistreptococcic serum in cases of human infection: in two instances in poisoning, once from a cadaver; in ninety-nine instances of pronounced puerperal fever; in nineteen cases of infection following operations, and in a large number of cases of erysipelas. The results were most satisfactory. Of the ninety-nine cases of puerperal fever, two-thirds were saved. The cadaveric poisoning was apparently stopped at once.

As to the use of the medicament, from 250 to 300 grammes of the serum are injected within twenty-four hours into the subcutaneous tissues, and as near as possible to the seat of poisoning. The most important point in the treatment consists in administering the serum daily. It must be prepared fresh, lasting only from ten to fourteen days.—*Therapeutic Gazette.*

Historical Pharmacy. The first alkaloid to be isolated from any plant by modern chemistry was morphine, which was discovered in 1817 by the German apothecary, Freidrich Wilhelm. Serturner, strychnine, and brucine were discovered by MM. Pelletier and Caventou in 1818, although, according to George Ebers, the Egyptologist, strychnine was known and used as a criminal poison by the ancient Egyptian as far back as the reign of Rameses the great.

Thomas Fowler, the originator of Fowler's solution, was born at Stratford, England, in 1736, and was by profession an apothecary.

Harvey and the Circulation of the Blood.—Dr. Wm. Harvey worked out the circulation of the blood between the years 1616 and 1619. His great work entitled *Motion of the Heart and Blood* was published in 1628. This was a small quarto of seventy-two pages. According to the opinion of Dr. J. C. Dalton, this book "undoubtedly contained a greater amount of important material in small compass than any other medical work ever published."

Adulteration of Yellow Wax. Lawall and Pursel (*American Journal of Pharmacy*) recently examined twelve samples of yellow wax. Five of these were found adulterated with either paraffin or stearic acid, or both, and one with tallow and carnauba wax; only half the samples were pure and conformed to the pharmacopœial requirements.

